# E-cigarettes in college: Associations between mental health and e-cigarette use with other substances

**DOI:** 10.18332/tpc/188712

**Published:** 2024-05-31

**Authors:** Christine M. Kava, Shannon L. Watkins, Paul A. Gilbert, Tanya J. Villhauer, Trisha L. Welter, Rima A. Afifi

**Affiliations:** 1 Department of Health Systems and Population Health, University of Washington School of Public Health, Seattle, United States; 2 Department of Community and Behavioral Health, University of Iowa College of Public Health, Iowa City, United States; 3 The Office of the Dean of Students, University of Iowa, Iowa Memorial Union, Iowa City, United States; 4 Student Wellness, University of Iowa, Iowa City, United States

**Keywords:** electronic nicotine delivery systems, alcohol, cannabis, co-use, college

## Abstract

**INTRODUCTION:**

College students are a priority population for substance use prevention, and other studies have reported associations between mental health and e-cigarette use. This study described the association of mental health to e-cigarette and other substance use (ECIG+ use) among US college students.

**METHODS:**

We used Fall 2018 and Spring 2019 National College Health Assessment data among undergraduate students aged 18–24 years (n=55654) at 138 institutions. We characterized substance use patterns and used multinomial regression to model adjusted odds of past 30-day ECIG use type [no substance use (reference); sole e-cigarette use; e-cigarette use and other substance use (ECIG+ use); no e-cigarette use but other substance use] by mental health characteristics, past 12-month diagnosis/treatment and psychological distress, individual characteristics, and college characteristics.

**RESULTS:**

Alcohol was the most prevalent substance (58%) used, followed by cannabis (23%) and e-cigarettes (15%). Nearly all (95%) students who used e-cigarettes reported using another substance. Adjusted odds of ECIG+ use (vs no substance use) were higher among students with past 12-month mental health diagnosis/treatment (AOR=1.5; 95% CI: 1.4–1.6) and higher psychological distress (AOR=1.1; 95% CI: 1.1–1.2). Other characteristics significantly associated with ECIG+ use included gender identity, sexual orientation, race and ethnicity, self-rated health, year in school, cumulative grade average, fraternity/sorority membership, and current residence.

**CONCLUSIONS:**

Most students who used e-cigarettes also reported other substance use, and this pattern of use was associated with poorer mental health outcomes than no substance use. Clarifying the relationship between mental health and ECIG+ use may enhance health interventions for college students.

## INTRODUCTION

In the past decade, young adults have been leading adopters of e-cigarettes, which are electronic devices that aerosolize a nicotine-containing liquid that is inhaled. In 2018, the percentage of adults aged 18–24 years who had ever used an e-cigarette was 26% compared to 21%, 11%, and 5% among adults aged 25–44, 45–64, and >65 years, respectively^1^. More recent pod-style devices appeal to young people because of their easy concealment, flavors, novel technology, and perceived safety relative to cigarettes^2^. Concerns about e-cigarette use by young people include negative impacts on the developing brain, exposure to toxicants in e-cigarette aerosol, and increased risk of using combustible cigarettes and other substances^2^.

The transition from adolescence to early adulthood is an important developmental stage that can include major life changes such as leaving a parental home, identity development, social group formation, and behavioral experimentation. For young adults who attend college, college’s unique social environment may promote substance use, including e-cigarette use, through social network influence^3^ and (mis)perceived norms of substance use among peers^4^. Indeed, college students report that friends influence their e-cigarette initiation^5^, report commonly using e-cigarettes with others and at social occasions^6^, and perceive high acceptability of e-cigarette use among peers^5,7,8^.

The use of multiple substances is common among young adults^9^, and the college environment might contribute to use. While past 30-day use of e-cigarettes is higher among young adults who do not attend versus attend college (15% vs 10%, respectively), among young adults who use e-cigarettes, the prevalence of multiple substance use is higher among college young adults^7^. Risks of using multiple substances include worsened side effects (e.g. blackouts), heightened risk of overuse and overdose, impaired judgment, and risky behaviors^10^. Long-term risks include addiction, related health consequences from all substances used, and worsened mental illness^11,12^. Multiple substance use might also encourage heavier use. For example, dual use of e-cigarettes and combustible cigarettes is associated with heavier use of both substances among college students^13^.

Several individual factors are associated with the use of multiple substances among college students, including age and identifying as White, male, sexual minority, and belonging to a college fraternity or sorority^14^. Previous studies conducted among young adults have found a greater likelihood of other substance use, including binge drinking and cannabis use, among those who use e-cigarettes compared to those who do not^15^. For example, adolescents and young adults who use e-cigarettes have higher odds of using cannabis^16^, and studies among youth have found an association between cannabis use and initiation of e-cigarette use^17^. E-cigarette use with and without other substances has not been well explored among college students.

Substance use among college students, including e-cigarette use, is associated with poor mental health (e.g. psychiatric disorders, high stress, depression)^18^. Many potential explanations exist for this relationship, including self-medication^19^ and nicotine’s negative impact on the developing brain in ways that undermine mental health, such as oxidative stress and emotional dysregulation^20^.

Although education level has a strong and consistently positive association with health outcomes, including substance use and mental health^21^, college also presents unique risks for substance use and mental distress. The prevalence of several mental health conditions among college students, including depression, anxiety, and suicidality, has been increasing^22^. As a result, college students are a priority population for both substance use prevention and mental health intervention.

Understanding the relationships between mental health distress and e-cigarette use is critical, given their increasing prevalence and potential intersections. The common use of e-cigarettes and other substances among young people makes it important to differentiate between patterns of e-cigarette use with and without the use of other substances and their associations with mental health. This study aims to: 1) describe the prevalence of use of both e-cigarettes and other substances (ECIG+ use); and 2) assess the relationship between mental health and ECIG+ use among college students.

## METHODS

### Design and sample

**Figure 1:**
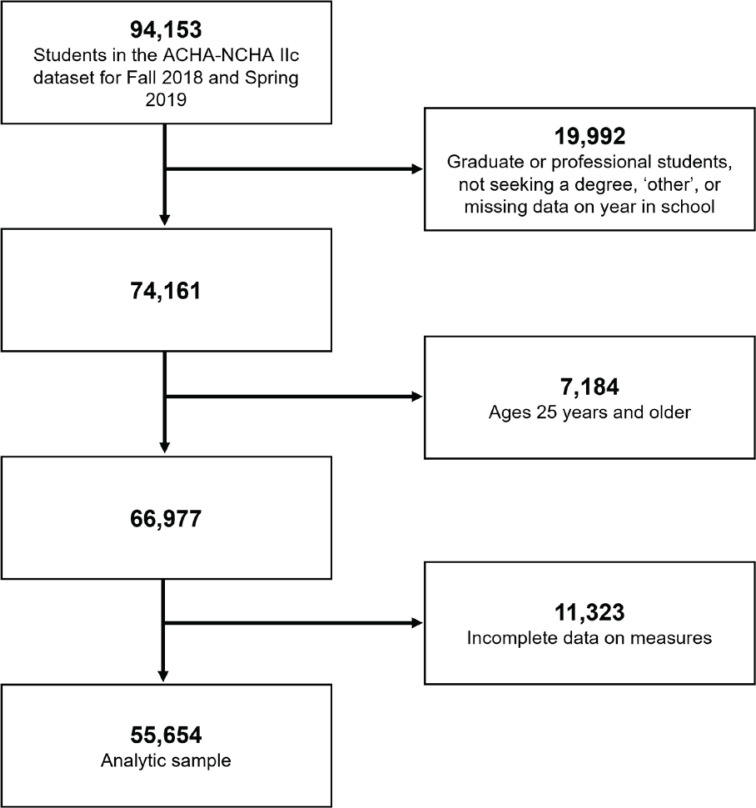
Study population flow chart, National College Health Assessment IIc, United States, Fall 2018–Spring 2019

This secondary analysis used data from the US American College Health Association’s National College Health Assessment (ACHA-NCHA IIc), a national survey of undergraduate and graduate students collected from 138 colleges who self-selected to participate in Fall 2018 and Spring 2019^23^. Only institutions that sampled all students or used random sampling techniques were included in the ACHA-NCHA public-use database. Reliability and validity analyses have demonstrated strong consistency over survey periods. We restricted our analysis to undergraduate students aged 18–24 years with complete data on our measures (Figure 1; n=55654). No Institutional Review Board approval was needed since the dataset is publicly available (with ACHA approval) and de-identified.

### Measures

*Substance use*

Our dependent variable was ECIG use type [1=no substance use (reference); 2=sole e-cigarette use; 3=e-cigarette use and other substance use (ECIG+ use); 4=no e-cigarette use but other substance use]. Students were asked to report how many days they used e-cigarettes within the last 30 days. Categorical response options ranged from 1=never used to 8=used daily. We classified past 30-day e-cigarette use as any versus none.

Regarding other substance use, students were asked to report, within the last 30 days, how many days they used alcohol, cannabis, other tobacco products, and illicit drugs (1=never used to 8=used daily). Other tobacco products included cigarettes, cigars, little cigars, clove cigarettes, hookah, and smokeless tobacco. Illicit drugs (excluding cannabis) included anabolic steroids, cocaine, hallucinogens, inhalants, MDMA, methamphetamine, opiates, other amphetamines, other club drugs, other illegal drugs, and sedatives. We coded students who reported using alcohol, cannabis, any other tobacco product, and any illicit drug at least one day within the past month as a past 30-day user of that substance category.

*Mental health status*

We combined variables to create two mental health indicators: psychological distress and mental health diagnosis/treatment. The psychological distress measure used 11 items assessing mental health symptoms. Example symptoms included feeling hopeless, overwhelmed, very sad, so depressed that it was difficult to function, and overwhelming anxiety. Categorical response options ranging from 1=no, never to 5=yes, in the last 12 months were recoded as dichotomous indicators of past 12-month symptoms (1=yes vs 0=no). We then summed all items to create an aggregate psychological distress measure, with higher scores denoting more distress symptoms within the past 12 months (range: 0–11).

Students were also asked to indicate within the past 12 months whether they had been diagnosed or treated by a professional for any of the following conditions: anorexia, anxiety, attention deficit and hyperactivity disorder, bipolar disorder, bulimia, depression, insomnia, other sleep disorder, obsessive-compulsive disorder, panic attacks, phobia, schizophrenia, substance use or addiction, other addiction (e.g. gambling, internet, sexual), or other mental health condition. We examined any past 12-month mental health diagnosis/treatment as a dichotomous indicator (1=yes vs 0=no). Since our dependent variable pertained to substance use, we excluded substance use disorders from this measure.

We included both mental health status variables in our analysis because they assessed different constructs and were not mutually exclusive. For example, students who experienced psychological distress may not have received a mental health diagnosis or sought treatment for their symptoms. Further, the psychological distress variable captures students with less severe mental health issues who experience distress but do not have a diagnosable illness. The relatively low correlation between psychological distress and mental health diagnosis/treatment (rpb=0.33) suggests that these variables contribute unique information.

*Student and college characteristics*

We included the following individual characteristics: age, gender identity, sexual orientation, race and ethnicity, self-rated health, year in school, cumulative grade average, and international student status. We included two variables considered to be individual-level proxies of their environment: social fraternity or sorority membership and location of current residence. At the college level, we examined institution type (private religious, private non-religious, or public), institution years (two-year vs four or more-year institution), and campus size (number of students enrolled).

### Data analysis

We conducted data analysis using Stata version 15.1 (Stata Corporation LLC, College Station, TX). First, we calculated descriptive statistics for all measures. Then, we characterized substance-use combinations with frequency counts and percentages among students using e-cigarettes and other substances. We ran bivariate statistics to estimate the associations between mental health status, individual characteristics, and ECIG use type. We set the critical alpha=0.05 and applied the Bonferroni correction to account for multiple comparisons.

We ran three multinomial logistic regression models to examine the adjusted associations between student, mental health, college characteristics, and ECIG use type. First, we estimated only the relationship between student characteristics and ECIG use type (Model 1); then, we included both student and mental health characteristics (Model 2); and finally, we included student, mental health, and college characteristics (Model 3). We used a nested approach to this analysis because we wanted to understand the added contribution of mental health to ECIG use type and how the association between mental health and ECIG use type might change after accounting for differences at the college level. We excluded age as a covariate to avoid multicollinearity, as our sample was restricted to students aged 18–24 years, and the year in school served as a proxy for age. We adjusted standard errors using the *vce* command in Stata to account for clustering at the college level. We calculated variance inflation factor (VIF) values to assess for multicollinearity among our independent variables and determined that there were no issues in our regression models.

We conducted three sensitivity analyses to further examine the relationships between student and college characteristics and ECIG use type. Specifically, we: 1) compared our analytic sample to eligible ACHA-NCHA participants (i.e. undergraduate students aged 18–24) who were excluded due to incomplete data on our measures; 2) examined bivariate associations between student and mental health characteristics; and 3) examined the association between sexual orientation and ECIG use type stratified by gender to elucidate our findings for sexual orientation.

## RESULTS

Majorities of the sample identified as cisgender women (68%), straight (80%), and White (59%) and attended four-year (91%) and public (66%) institutions (Table 1). Ninety percent of students reported having an A or B cumulative grade average. The mean psychological distress score was 5.6 (SD=2.9; range 0–11). About one-third (31%) of students had received a mental health diagnosis/treatment in the past 12 months. All bivariate associations between our individual characteristics and ECIG use type were statistically significant after applying the Bonferroni correction (Supplementary file Table S1).

**Table 1:** Participant characteristics, National College Health Assessment IIc, United States, Fall 2018–Spring 2019 (N=55654)

*Characteristics*	*n*	*%*
**Sociodemographic**		
**Age** (years), mean (SD)	19.94	1.50
**Gender identity**		
Cisgender woman	37986	68.25
Cisgender man	16039	28.82
Transgender, genderqueer, or other identity^a^	1629	2.93
**Sexual orientation**		
Straight	44763	80.43
Gay/lesbian	1756	3.16
Bisexual	5303	9.53
Other^b^	3832	6.89
**Race and ethnicity**		
White	32987	59.27
Hispanic or Latino	6243	11.22
Black	2392	4.30
Asian or Pacific Islander	7145	12.84
Multi-race or other^c^	6887	12.37
**Self-rated health**		
Excellent	6185	11.11
Very good	19870	35.70
Good	19541	35.11
Fair	8789	15.79
Poor	1269	2.28
**Year in school**		
First	17004	30.55
Second	13626	24.48
Third	13036	23.42
Fourth	10124	18.19
Fifth or higher	1864	3.35
**Cumulative grade average**		
A or B grades	49892	89.65
C grade or lower	5762	10.35
International student	2561	4.60
Member of social fraternity or sorority	5495	9.87
**Current residence^d^**		
On-campus housing	28166	50.61
Fraternity or sorority house	622	1.12
Off-campus housing	25701	46.18
Other	1165	2.09
**Psychological distress score^e^,** mean (SD)	5.56	2.85
**Mental health diagnosis/treatment in past 12 months**	17341	31.16
**College characteristics** (N=138)		
**Study period**		
Fall 2018	40	28.99
Spring 2019	98	71.01
**Institution type**		
Private religious	24	17.39
Private non-religious	23	16.67
Public	91	65.94
**Institution years**		
2	13	9.42
≥4	125	90.58
**Campus size**		
<2500	29	21.01
2500–4999	15	10.87
5000–9999	23	16.67
10000–19999	32	23.19
≥20000	39	28.26

a Transgender is an umbrella term to describe people whose gender identity differs from their sex assigned at birth, which is usually based on visible anatomical characteristics (e.g. genitalia). In contrast, cisgender refers to people whose gender identity aligns with sex assigned at birth. Genderqueer refers to people whose gender identify falls outside of, in between, or fluctuates among binary gender categories (man and woman). Transgender people may also identify as non-binary, which could include people who identify as genderqueer, both male and female (bigender), neither gender (agender), or experience their gender fluidly within the gender spectrum (gender-fluid).

b Includes asexual, pansexual, queer, questioning, same gender loving, and another identity.

c Includes American Indian, Alaskan Native, or Native Hawaiian; biracial or multiracial; and other race.

d On-campus housing includes campus residence hall and other college/university housing. Off-campus housing includes parent/guardian’s home and other off-campus housing.

e Scores range from 0–11, with higher scores indicating a greater number of distress symptoms within the past 12 months.

**Figure 2:**
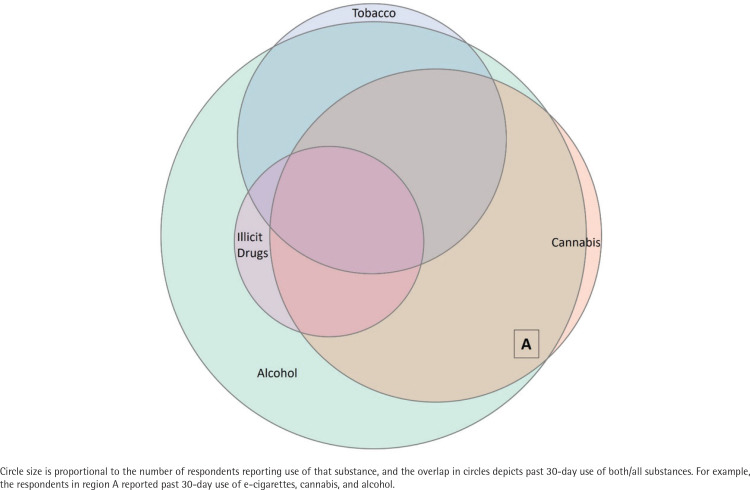
Top substance use combinations among students who used e-cigarettes and other substances, National College Health Assessment IIc, United States, Fall 2018–Spring 2019 (N=7987)

Table 2 reports the prevalence of past 30-day substance use in our sample. Alcohol was the most used substance (58%), followed by cannabis (23%) and e-cigarettes (15%). Among those who reported past 30-day e-cigarette use, nearly all (95%) reported also using at least one other substance; alcohol appeared in several use patterns, and the most common unique substance use combination was e-cigarette, alcohol, and cannabis use (26%). Only 2% of these students used e-cigarettes with other tobacco products only. Figure 2 displays the top substance use combinations among students who used e-cigarettes and other substances based on the ten combinations with prevalence ≥1%. Circle size is proportional to the number of respondents reporting the use of that substance, and overlapping circles indicate substance use combinations.

**Table 2:** Substance use, National College Health Assessment IIc, United States, Fall 2018–Spring 2019, ACHA-NCHA (N=55654)

*Consumption*	*n*	*%*
**Past 30-day substance use^a^**		
E-cigarettes	8437	15.16
Alcohol	32153	57.77
Cannabis	12847	23.08
Cigarettes	3249	5.84
Illicit drugs^b^	3002	5.39
Other tobacco^c^	2944	5.29
**E-cigarette and other substance use**		
No substance use	21264	38.21
Sole e-cigarette use	450	0.81
E-cigarette use and other substance use	7987	14.35
No e-cigarette use but other substance use	25953	46.63
**Substance combinations among students who used e-cigarettes and other substances (N=7987)^d^**		
E-cigarettes + alcohol + cannabis	2060	25.79
E-cigarettes + alcohol	2054	25.72
E-cigarettes + alcohol + cannabis + tobacco	1183	14.81
E-cigarettes + alcohol + tobacco	818	10.24
E-cigarettes + alcohol + cannabis + tobacco + illicit drugs	759	9.50
E-cigarettes + alcohol + cannabis + illicit drugs	498	6.24
E-cigarettes + cannabis	134	1.68
E-cigarettes + tobacco	129	1.62
E-cigarettes + alcohol + tobacco + illicit drugs	125	1.57
E-cigarettes + alcohol + illicit drugs	114	1.43
E-cigarettes + cannabis + tobacco	48	0.60
E-cigarettes + cannabis + illicit drugs	29	0.36
E-cigarettes + cannabis + tobacco + illicit drugs	17	0.21
E-cigarettes + illicit drugs	12	0.15
E-cigarettes + tobacco + illicit drugs	7	0.09

a Not mutually exclusive.

b Illicit drugs include cocaine, methamphetamine, other amphetamines, sedatives, hallucinogens, anabolic steroids, opiates, inhalants, MDMA, other club drugs, and other illegal drugs.

c Other tobacco includes hookah, cigars, little cigars, and clove cigarettes, and smokeless tobacco.

d Tobacco includes both cigarettes and other tobacco products.

Table 3 displays the results from our multinomial logistic regression models. In our fully specified model, greater psychological distress was associated with higher odds of ECIG+ use versus no substance use (AOR=1.1; 95% CI: 1.1–1.2) and higher odds of no e-cigarette use but other substance use (AOR=1.1; 95% CI: 1.1–1.2). The association between psychological distress and sole e-cigarette use was not significant. Receiving a mental health diagnosis/treatment in the past 12 months was associated with higher odds of sole e-cigarette use (AOR=1.6; 95% CI: 1.3–2.0), ECIG+ use (AOR=1.5; 95% CI: 1.4–1.6), and no e-cigarette use but other substance use (AOR=1.1; 95% CI: 1.1– 1.2) compared to no substance use. Our findings for mental health were the same in our model accounting for only student and mental health characteristics.

**Table 3:** Multinomial logistic regression^a^, National College Health Assessment IIc, United States, Fall 2018–Spring 2019 (N=55654)

*Variables*	*Model 1: Student characteristics*			*Model 2: Student + mental health characteristics*			*Model 3: Student + mental health + college characteristics*		
	*Sole e-cigarette use*	*E-cigarette use and other substance use*	*No e-cigarette use but other substance use*	*Sole e-cigarette use*	*E-cigarette use and other substance use*	*No e-cigarette use but other substance use*	*Sole e-cigarette use*	*E-cigarette use and other substance use*	*No e-cigarette use but other substance use*
	*AOR (95% CI)*	*AOR (95% CI)*	*AOR (95% CI)*	*AOR (95% CI)*	*AOR (95% CI)*	*AOR (95% CI)*	*AOR (95% CI)*	*AOR (95% CI)*	*AOR (95% CI)*
**Gender identity^b^** (ref: Cis woman)									
Cis man	**1.42 (1.14–1.77)**	**1.47 (1.33–1.61)**	**0.78 (0.72–0.84)**	**1.55 (1.26–1.91)**	**1.73 (1.57–1.90)**	**0.85 (0.79–0.91)**	**1.55 (1.26–1.91)**	**1.73 (1.58–1.90)**	**0.84 (0.79–0.90)**
Transgender, genderqueer, or other identity	1.50 (0.90–2.48)	0.94 (0.81–1.09)	**0.82 (1.72–0.94)**	1.40 (0.84–2.33)	**0.86 (0.73–1.00)**	**0.79 (0.69–0.90)**	1.41 (0.84–2.36)	**0.86 (0.74–1.00)**	**0.78 (0.69–0.89)**
**Sexual orientation** (ref: Straight)									
Gay/lesbian	1.21 (0.63–2.33)	**1.29 (1.10–1.51)**	**1.61 (5.72–1.84)**	1.09 (0.57–2.11)	1.03 (0.88–1.22)	**1.44 (1.25–1.66)**	1.15 (0.61–2.20)	1.02 (0.87–1.20)	**1.40 (1.22–1.61)**
Bisexual	**1.63 (1.22–2.19)**	**2.09 (1.88–2.32)**	**1.74 (0.72–1.90)**	**1.45 (1.08–1.95)**	**1.65 (1.48–1.83)**	**1.54 (1.41–1.68)**	**1.47 (1.10–1.98)**	**1.65 (1.49–1.83)**	**1.52 (1.40–1.66)**
Other sexual orientation^c^	0.86 (0.57–1.31)	1.06 (0.90–1.23)	1.08 (4.72–1.21)	0.78 (0.51–1.20)	0.85 (0.73–1.00)	0.97 (0.87–1.08)	0.81 (0.53–1.24)	0.85 (0.73–1.00)	0.94 (0.85–1.05)
**Race and ethnicity** (ref: White)									
Hispanic or Latino	**0.34 (0.24–0.48)**	**0.35 (0.27–0.45)**	**0.54 (0.72–0.65)**	**0.37 (0.26–0.53)**	**0.37 (0.28–0.48)**	**0.55 (0.45–0.66)**	**0.43 (0.31–0.60)**	**0.35 (0.28–0.43)**	**0.51 (0.43–0.59)**
Black	**0.19 (0.09–0.38)**	**0.17 (0.14–0.21)**	**0.58 (0.72–0.67)**	**0.20 (0.10–0.41)**	**0.18 (0.15–0.23)**	**0.59 (0.51–0.69)**	**0.21 (0.10–0.42)**	**0.19 (0.15–0.23)**	**0.61 (0.51–0.72)**
Asian or Pacific Islander	**0.29 (0.18–0.45)**	**0.34 (0.28–0.42)**	**0.76 (0.72–0.88)**	**0.31 (0.20–0.48)**	**0.37 (0.30–0.46)**	**0.78 (0.67–0.91)**	**0.35 (0.22–0.54)**	**0.38 (0.32–0.46)**	**0.78 (0.68–0.89)**
Multi-race or other^d^	0.88 (0.66–1.17)	**0.74 (0.63–0.85)**	0.90 (3.72–1.02)	0.89 (0.67–1.19)	**0.73 (0.62–0.84)**	0.89 (0.78–1.01)	0.95 (0.71–1.26)	**0.71 (0.62–0.81)**	**0.86 (0.77–0.96)**
**Self-rated health** (ref: Excellent)									
Very good	1.16 (0.83–1.61)	**1.48 (1.35–1.62)**	**1.28 (3.72–1.37)**	1.11 (0.79–1.54)	**1.29 (1.17–1.41)**	**1.19 (1.11–1.27)**	1.10 (0.79–1.53)	**1.31 (1.19–1.43)**	**1.21 (1.13–1.29)**
Good	1.38 (0.98–1.95)	**1.78 (1.61–1.98)**	**1.26 (0.72–1.35)**	1.25 (0.88–1.76)	**1.35 (1.21–1.50)**	**1.08 (1.01–1.16)**	1.23 (0.87–1.74)	**1.38 (1.23–1.54)**	**1.11 (1.04–1.20)**
Fair	**1.46 (1.00–2.14)**	**1.75 (1.56–1.96)**	**1.14 (0.72–1.25)**	1.26 (0.85–1.85)	**1.17 (1.04–1.32)**	0.92 (0.85–1.01)	1.24 (0.84–1.84)	**1.20 (1.06–1.36)**	0.95 (0.87–1.03)
Poor	1.54 (0.86–2.76)	**1.98 (1.60–2.45)**	1.04 (1.72–1.21)	1.23 (0.68–2.21)	1.14 (0.93–1.41)	**0.77 (0.66–0.90)**	1.25 (0.69–2.25)	1.16 (0.94–1.43)	**0.78 (0.66–0.91)**
**Year in school** (ref: First year)									
Second	**0.58 (0.47–0.72)**	1.04 (0.95–1.14)	**1.46 (0.72–1.56)**	**0.58 (0.47–0.72)**	1.02 (0.93–1.11)	**1.44 (1.35–1.54)**	**0.59 (0.47–0.73)**	0.99 (0.90–1.09)	**1.40 (1.31–1.49)**
Third	**0.43 (0.32–0.59)**	**1.45 (1.29–1.63)**	**2.81 (0.72–3.08)**	**0.43 (0.32–0.58)**	**1.42 (1.26–1.60)**	**2.78 (2.53–3.06)**	**0.43 (0.32–0.59)**	**1.36 (1.20–1.53)**	**2.67 (2.42–2.95)**
Fourth	**0.40 (0.27–0.60)**	**2.08 (1.82–2.38)**	**5.19 (0.72–5.77)**	**0.40 (0.27–0.59)**	**2.07 (1.82–2.37)**	**5.20 (4.67–5.78)**	**0.40 (0.27–0.60)**	**1.94 (1.70–2.21)**	**4.88 (4.38–5.44)**
Fifth or more	**0.48 (0.23–0.98)**	**1.68 (1.41–2.01)**	**4.36 (0.72–5.11)**	**0.46 (0.22–0.94)**	**1.61 (1.35–1.93)**	**4.31 (3.68–5.04)**	**0.46 (0.22–0.94)**	**1.56 (1.31–1.87)**	**4.32 (3.66–5.11)**
**Cumulative grade average** (ref: A or B grades)									
C grade or lower	**2.70 (2.09–3.47)**	**1.59 (1.41–1.80)**	1.05 (0.72–1.13)	**2.58 (2.01–3.31)**	**1.46 (1.29–1.65)**	1.00 (0.93–1.09)	**2.41 (1.87–3.09)**	**1.53 (1.35–1.73)**	1.07 (0.98–1.15)
**International student** (ref: No)									
Yes	1.07 (0.68–1.69)	0.97 (0.72–1.29)	0.90 (7.72–1.07)	1.09 (0.69–1.70)	0.99 (0.74–1.34)	0.92 (0.77–1.09)	1.14 (0.74–1.76)	0.96 (0.72–1.27)	0.87 (0.74–1.03)
**Member of social fraternity or sorority** (ref: No)									
Yes	**2.05 (1.37–3.06)**	**5.99 (5.21–6.88)**	**3.01 (0.72–3.39)**	**2.03 (1.36–3.03)**	**6.02 (5.22–6.94)**	**3.02 (2.67–3.42)**	**2.10 (1.40–3.15)**	**5.78 (5.05–6.63)**	**2.93 (2.62–3.27)**
**Current residence^e^** (ref: On-campus housing)									
Fraternity or sorority house	1.83 (0.75–4.46)	1.51 (0.98–2.32)	1.01 (1.72–1.43)	1.86 (0.76–4.56)	**1.59 (1.04–2.44)**	1.04 (0.74–1.47)	1.86 (0.75–4.63)	**1.68 (1.11–2.55)**	1.11 (0.80–1.54)
Off-campus housing	1.04 (0.80–1.35)	1.00 (0.84–1.20)	**0.79 (7.72–0.90)**	1.04 (0.80–1.35)	1.00 (0.83–1.20)	**0.79 (0.69–0.91)**	1.02 (0.78–1.33)	1.11 (0.93–1.33)	0.89 (0.78–1.01)
Other residence	1.65 (0.89–3.04)	1.26 (0.95–1.68)	1.11 (1.72–1.43)	1.60 (0.86–2.96)	1.26 (0.94–1.68)	1.11 (0.86–1.43)	1.52 (0.80–2.91)	**1.44 (1.06–1.95)**	1.26 (0.97–1.64)
**Psychological distress score^f^**				1.02 (0.98–1.07)	**1.14 (1.12–1.15)**	**1.08 (1.07–1.09)**	1.03 (0.99–1.07)	**1.13 (1.12–1.15)**	**1.08 (1.07–1.09)**
**Mental health diagnosis/treatment in past 12 months** (ref: No)									
Yes				**1.63 (1.32–2.02)**	**1.47 (1.37–1.57)**	**1.13 (1.07–1.20)**	**1.63 (1.32–2.01)**	**1.47 (1.38–1.58)**	**1.13 (1.07–1.20)**
**Institution type** (ref: Private religious)									
Private non-religious							0.55 (0.30–1.00)	1.09 (0.75–1.58)	1.28 (0.97–1.70)
Public							1.25 (0.85–1.82)	0.71 (0.49–1.02)	**0.68 (0.51–0.91)**
**Institution years** (ref: Two year)									
Four or more years							1.35 (0.83–2.20)	1.35 (0.93–1.97)	1.00 (0.80–1.24)
**Campus size** (ref: <2500)									
2500–4999							1.06 (0.61–1.84)	1.29 (0.82–2.01)	1.02 (0.75–1.39)
5000–9999							0.70 (0.46–1.07)	1.34 (0.93–1.95)	1.21 (0.90–1.62)
10000–19999							0.74 (0.50–1.11)	1.26 (0.83–1.93)	1.11 (0.81–1.52)
≥20000							**0.58 (0.37–0.91)**	1.52 (0.98–2.37)	**1.11 (0.81–1.52)**
**Study period** (ref: Fall 2018)									
Spring 2019	0.96 (0.68–1.35)	0.84 (0.65–1.08)	0.89 (8.72–1.10)	0.95 (0.68–1.33)	0.82 (0.64–1.06)	0.88 (0.71–1.08)	0.97 (0.73–1.29)	0.87 (0.69–1.10)	0.96 (0.80–1.14)

a Reference category is no substance use. Bold font indicates statistical significance.

b Transgender is an umbrella term to describe people whose gender identity differs from their sex assigned at birth, which is usually based on visible anatomical characteristics (e.g. genitalia). In contrast, cisgender refers to people whose gender identity aligns with sex assigned at birth. Genderqueer refers to people whose gender identify falls outside of, in between, or fluctuates among binary gender categories (man and woman). Transgender people may also identify as non-binary, which could include people who identify as genderqueer, both male and female (bigender), neither gender (agender), or experience their gender fluidly within the gender spectrum (gender-fluid).

c Includes asexual, pansexual, queer, questioning, same gender loving, and another identity.

d Includes American Indian, Alaskan Native, or Native Hawaiian, biracial or multiracial, and other race.

e On-campus housing includes campus residence hall and other college/ university housing. Off-campus housing includes parent/guardian’s home and other off-campus housing.

f Scores range from 0–11, with higher scores indicating a greater number of distress symptoms within the past 12 months.

Several student characteristics were significantly associated with ECIG use type, including gender identity, sexual orientation, race and ethnicity, self-rated health, year in school, cumulative grade average, social fraternity or sorority membership, and current residence. For example, compared to White students, students of all other racial and ethnic identities had lower odds of ECIG+ use versus no substance use (AORs range: 0.51–0.86). The associations between student characteristics and ECIG use type were generally similar across the three models, although some findings changed in statistical significance when accounting for mental health and college characteristics.

Attending a college with a campus size of ≥20000 versus <2500 was associated with lower odds of sole e-cigarette use versus no substance use (AOR=0.58; 95% CI: 0.37–0.91). In contrast, the odds of no e-cigarette use but other substance use were higher among students on campuses of ≥20000 (AOR=1.11; 95% CI: 0.81–1.52). Students who attended a public versus private, non-religious institution had lower odds of no e-cigarette use but other substance use versus no substance use (AOR=0.68; 95% CI: 0.51–0.91). No college characteristics were significantly associated with ECIG+ use.

### Sensitivity analysis

When comparing our analytic sample to eligible participants excluded due to incomplete data (n=11323), there were significant differences in ECIG use type but not psychological distress and mental health diagnosis/treatment. We found significant differences between the two samples across all student characteristics except for current residence. The percentage of missing data on our measures among excluded participants ranged from <1% for current residence to 34% for mental health diagnosis/treatment, though most variables had <10% missing data.

In our stratified analyses by gender, cisgender women who identified as bisexual (vs straight) had higher odds of belonging to each of the three substance use categories (vs no substance use) (AORs=1.6–2.2). Cisgender women who identified as lesbian also had higher odds of no e-cigarette use but other substance use versus no substance use (AOR=1.3; 95% CI: 1.1–1.6). For cisgender men, odds of ECIG+ use and no e-cigarette but other substance use were higher among gay and bisexual students (AORs=1.3–1.8). For transgender, genderqueer, or other identity students, the odds of no e-cigarette but other substance use versus no substance use was higher among gay/lesbian and bisexual students (AORs=1.6–1.9).

## DISCUSSION

This study investigated the prevalence and correlates of ECIG+ use among college students, a priority population for both substance use prevention and mental health support. Notable strengths include the use of a large national sample, our focus on multiple substances, and the inclusion of mental health variables, all of which may enhance understanding of college students’ substance use. Our analysis identified key patterns that help us better understand e-cigarette use among college students. While psychological distress was not associated with sole ECIG use, both psychological distress and past-year mental health diagnosis/treatment were positively associated with ECIG+ use versus no substance use. This latter finding is consistent with growing evidence of the association between e-cigarette use and mental health outcomes^18,24^.

Consistent with a previous study on adolescents^25^, we found that sole e-cigarette use was rare among college students and that nearly all students who used e-cigarettes also reported past 30-day use of another substance. Among other substances reported, alcohol, cannabis, and tobacco were the most common. Very few students who used e-cigarettes used only other nicotine products. Further, substance-use combinations, including cannabis (59%), were more common than combinations including tobacco (39%). This finding relates to the increasing popularity of cannabis; according to data from the Monitoring the Future panel survey, between 2017 and 2019, the past 30-day prevalence of cannabis use increased from 5% to 14% among college students^26^.

Previous studies of college students have identified unique behavioral patterns of multiple substance use^27^, suggesting that a variety of interventions may be necessary to reduce substance use among college students. For example, illicit drugs accounted for a relatively small share of substance use; however, use that includes illicit drugs may be especially dangerous and important to address. Identifying students who have particularly high-risk substance use and delivering tailored interventions should remain a part of the repertoire of campus responses.

Interventions incorporating technology (e.g. text messages, phone calls, online resources) show promise in improving mental health outcomes among college students; many of these interventions use evidence-based approaches such as cognitive behavioral therapy^28^. Prior studies have emphasized the importance of integrated care for mental health and substance use disorders^29^, so we recommend that both mental health symptoms and e-cigarette use be considered when designing treatment interventions for college students.

Interventions in the policy and regulatory environment may offer more impact than individual-level interventions. For example, campus-wide bans on tobacco products and vaping, campus or city restrictions on alcohol outlet density, and local or state regulation of commercial cannabis may reduce college students’ substance use. Given that <40% of campuses have adopted an e-cigarette-free policy and only 20% meet the American College Health Association recommendations for tobacco-free policies^30^, there is room for additional action on campus to deter substance use.

At present, the evidence-based interventions (EBIs) for use with college students are better developed for alcohol than other substances. Fewer EBIs have been developed for college students to prevent e-cigarette, cannabis, and other substance use. Thus, we see an opportunity to adapt and test existing tools, such as the Truth Initiative’s ‘This is Quitting’ program designed for teenagers and young adults^31^ to reduce substance use and improve mental health outcomes among college students.

Our analysis also suggests that there are certain demographic groups that warrant focused attention. Consistent with prior studies^13,25^, we found that cisgender males were more likely to report sole e-cigarette use and ECIG+ use versus no substance use than cisgender female students. A research report published by the National Institute on Drug Abuse describes several reasons why males may be more likely to use tobacco products, such as greater activation of reward pathways, greater responsiveness to environmental cues, and cultural factors^32^. Regardless of mechanisms, high-use prevalence suggests the need to tailor substance use prevention interventions for males.

Racial and ethnic minority students had consistently lower odds of ECIG+ use versus no substance use than their White peers. This finding may reflect a previously documented pattern of later age of substance-use initiation among these groups^33^. If so, prevention efforts in college settings may be an important strategy to address lifetime substance use disparities. There may also be untapped factors that could be leveraged in the college context, such as racial and ethnic community affiliation for health promotion.

Our sensitivity analyses suggest that higher ECIG+ use versus no substance use among bisexual females and males may be driving our findings of sexual orientation differences. This is consistent with prior studies showing a higher prevalence of substance use among bisexual individuals when compared to both heterosexual peers and other sexual minority groups^34^. In a recent study characterizing substance use disparities, bisexual women, in particular, were at an elevated risk for multiple substance use behaviors^34^, suggesting that this group may need focused and culturally appropriate prevention interventions.

Interestingly, no college characteristics were associated with ECIG+ use versus no substance use. However, students at public institutions had lower odds of no e-cigarette use but other substance use than students at private religious institutions. Larger campus size was also associated with lower odds of sole e-cigarette use and higher odds of no e-cigarette but other substance use. Differences in the social context and/or student body composition might help explain this result, though additional research is needed to fully understand these differences.

The high prevalence of other substance use among students who used e-cigarettes warrants an in-depth investigation into the motivations and consequences of this pattern of use. Potential motivations might include a desire to enhance positive subjective effects, physiological triggers for additional use, experimentation, and desire to reduce the use of one substance^35^. Further, shared devices may facilitate e-cigarette and cannabis use, but less is known about shared delivery mechanisms for illicit drugs other than cannabis.

Emerging research to identify physiological impacts, such as the toxicant load of dual-use (e.g. e-cigarette and combustible tobacco or alcohol), is important. Understanding the most appealing substance-use combinations, their motivations, and their health impacts could enhance the impact of prevention efforts for e-cigarettes by recognizing their frequent use of other substances. Continued exploration of the relationship and directionality between mental health and ECIG+ use is also critical to developing supportive and effective interventions.

### Strengths and limitations

Our findings should be interpreted in the context of strengths and limitations. Using a national sample of students at 138 US colleges allowed us to account for variations in college characteristics and culture. Since the ACHA-NCHA is cross-sectional, we could not determine whether poor mental health precipitated the use of e-cigarettes and other substances, whether this use caused erosion of mental health, or both. Longitudinal studies demonstrate unidirectional and bidirectional relationships between mental health and e-cigarette use^36^. Additional research is needed to establish and understand the mechanisms for causality.

Some demographic groups were over-represented (e.g. cisgender women) or under-represented (e.g. Black students) in the sample. This reduces our ability to explore sub-group differences and may constrain the generalizability of findings. The ACHA-NCHA used self-report measures, which may have been subject to reporting biases, particularly because substance use and mental health are sensitive and stigmatizing topics. Some important aspects of substance use were not assessed. For example, we could not investigate the sequencing, frequency, or intensity of substance use. We look forward to future studies that include a broader range of variables and longitudinal data that will be able to distinguish sequential versus concurrent multiple substance use.

## CONCLUSIONS

This study provides insights about ECIG+ use, among a large sample of college students coming from 138 institutions that may inform future research and practice. Nearly all college students who reported using e-cigarettes also reported using at least one other substance. Therefore, intervention efforts to reduce e-cigarette use should consider the role of these other substances. It is also important to consider poor mental health, which was associated with higher odds of ECIG+ use versus no substance use. While additional research is needed to clarify the direction of this relationship, developing interventions that can both address substance use and enhance mental health, and that are tailored to groups that experience the highest rates of ECIG+ use, may have the greatest impact on college student health.

## Supplementary Material



## Data Availability

The data supporting this research are available from the authors on reasonable request.
