# Supplementary material for: E-cigarettes in college: Associations between mental health and e-cigarette use with other substances
Source: Tob Prev Cessat. 2024 May 31;10:10.18332/tpc/188712. doi: 10.18332/tpc/188712 (PMC11141061; doi:10.18332/tpc/188712)
Supplement: Supplementary file 1 [file TPC-10-24-s1.pdf]

Table S1. Bivariate Statistics, National College Health Assessment IIc, United States, Fall 2018–Spring 2019 (n=55,654 students)

Variable	E-cigarette and other substance use (%)				P-Value
	No substance use	Sole e-cigarette use	E-cigarette use and other substance use	No e-cigarette use but other substance use	
Age (Mean)	19.59	19.18	19.87	20.26	0.000
Gender identity[a]					
Cis woman	66.17	58.44	61.09	72.34	
Cis man	30.90	37.11	36.08	24.74	0.000
Transgender, genderqueer, or another identity	2.93	4.44	2.83	2.92	
Sexual orientation					
Straight	83.39	78.89	78.63	78.58	
Gay/lesbian	2.63	3.33	3.11	3.60	0.000
Bisexual	6.87	11.33	12.07	10.89	
Other sexual orientation[b]	7.11	6.44	6.20	6.92	
Race and ethnicity					
White	51.22	68.44	71.97	61.80	
Hispanic or Latino/a	13.21	6.22	6.40	11.15	
Black	5.86	1.78	1.39	3.95	0.000
Asian or Pacific Islander	17.56	8.00	7.85	10.59	
Multi/other race[c]	12.14	15.56	12.40	12.50	
Self-rated health					
Excellent	13.16	10.44	8.64	10.21	
Very good	34.79	31.78	33.88	37.08	
Good	33.65	36.00	37.57	35.54	0.000
Fair	16.14	18.67	16.91	15.12	
Poor	2.26	3.11	2.99	2.06	
Year in school					
First	40.54	56.22	31.26	21.70	
Second	28.03	24.00	24.72	21.52	
Third	19.35	12.22	23.40	26.96	0.000
Fourth	9.90	5.78	17.50	25.41	
Fifth or more	2.18	1.78	3.12	4.41	
International student					
Cumulative grade average	89.91	79.56	87.08	90.39	0.000
A/B grades	10.09	20.44	12.92	9.61	
C grades or below	5.74	4.00	3.57	4.00	0.000
Member of social fraternity or sorority	3.64	7.11	20.28	11.82	0.000

Current residence[d]					
On-campus housing	54.43	59.78	49.15	47.77	0.000
Fraternity or sorority house	0.40	1.11	2.73	1.21	
Off-campus housing	43.52	36.67	45.99	48.58	
Other residence	1.66	2.44	2.13	2.44	
Psychological distress score[e] (Mean)	5.09	5.57	6.18	5.76	0.000
Mental health diagnosis/treatment in past 12 months	24.70	39.56	41.04	33.26	0.000

We used chi-square values based on univariate regression to calculate P-values.

[a]Transgender is an umbrella term to describe people whose gender identity differs from their sex assigned at birth, which is usually based on visible anatomical characteristics (e.g. genitalia)). In contrast, cisgender refers to people whose gender identity aligns with sex assigned at birth. Genderqueer refers to people whose gender identify falls outside of, in between, or fluctuates among binary gender categories (man and woman). Transgender people may also identify as non-binary, which could include people who identify as genderqueer, both male and female (bigender), neither gender (agender), or experience their gender fluidly within the gender spectrum (gender-fluid).

[b]Includes asexual; pansexual; queer; questioning; same gender loving; and another identity.

[c]Includes American Indian, Alaskan Native, or Native Hawaiian; biracial or multiracial; and other race.

[d]On-campus housing includes campus residence hall and other college/university housing. Off-campus housing includes parent/guardian's home and other off-campus housing.

[e]Scores range from 0-11, with higher scores indicating a greater number of distress symptoms within the past 12 months.

© 2024 Kava C. M. et al.
